# Transactional Links between Teacher–Adolescent Support, Relatedness, and Aggression at School: A Three-Wave Longitudinal Study

**DOI:** 10.3390/ijerph18020436

**Published:** 2021-01-07

**Authors:** Teresa I. Jiménez, Jaime León, José Martín-Albo, Andrés S. Lombas, Sonsoles Valdivia-Salas, Estefanía Estévez

**Affiliations:** 1 Department of Psychology and Sociology, University of Zaragoza, Ciudad Escolar s/n, 44003 Teruel, Spain; jmartina@unizar.es (J.M.-A.); slombas@unizar.es (A.S.L.); sonsoval@unizar.es (S.V.-S.); 2 Faculty of Teacher Training, University of Las Palmas de Gran Canaria, Juana de Arco, 1, 35004 Las Palmas de Gran Canaria, Spain; jaime.leon@ulpgc.es; 3 Departament of Health Psychology, Miguel Hernández University, Avda. de la Universidad s/n, 03202 Alicante, Spain; eestevez@umh.es

**Keywords:** adolescence, school aggression, relational aggression, overt aggression, teacher support, relatedness

## Abstract

This study examines the reciprocal effects between two school-based relationships within the classroom—namely, perceived teacher support and relatedness with classmates—and school aggression (overt and relational) across two courses of secondary education. Participants were 654 adolescents (48% boys), who were assessed in three waves: first, at the beginning of the academic year (T0), second, at the end of the same academic year (T1), and third, at the beginning of the next academic year (T2) (*M*_age wave 1_ = 13.98 years). Autoregressive cross-lagged modeling was applied. Results show a protective effect of relatedness against relational aggression in both genders. Moreover, we observed a protective effect of perceived teacher support at the beginning of the course for later school aggression as well as a risk effect if this perceived teacher support is maintained throughout the course. These effects were observed in relation with gender-atypical forms of aggression (overt in girls and relational in boys). Finally, aggression had negative consequences for relatedness in girls and for teacher support through the mediation of relatedness in boys. Gender differences and practical implications of these findings are discussed.

## 1. Introduction

One of the challenges of the adolescent stage is the development of significant social relationships with peers and non-parental adults such as teachers [1]. Secondary schools are places where adolescents spend a significant amount of time learning with and from peers and teachers. Students who develop supportive relationships with teachers and feelings of relatedness with classmates are more likely to succeed academically and promote their well-being [2] than those with poor school-based relationships. However, one of the dark sides of social relationships in the school setting is the aggressive behavior exhibited by some adolescents toward their peers. Among the different acts involving school violence (i.e., aggression toward school staff, property damage, vandalism on the campus, and bullying), the current study focused on school aggression—that is, on behaviors aimed at other classmates to cause harm intentionally, at the physical, psychological, or relational level.

Research in the last two decades has identified aggression at secondary school as a serious problem in European [3], North American [4], Latin American [5], and Asian countries [6]. The relevance of this issue is related to the severity of the related outcomes at the personal (e.g., anxiety, depression, low self-esteem and life satisfaction), family (e.g., conflict, communication problems, low affective union), and school level (e.g., negative attitudes toward school, low academic achievement, school dropout) of this type of aggression for the victims as well as for the aggressors [7,8]. Most of the studies in this field have focused on analyzing risk factors that explain the development of school aggression [9], but fewer studies have accounted for its consequences [7]. Moreover, very few studies have focused on simultaneously analyzing the causes and consequences of school aggression at a relational level. Given the importance of the social school-based relationships with adults and peers needed to facilitate a successful passage through adolescence [1], the main objective of this study is to analyze the transactional links between social school-based relationships (assessed through perceived teacher–adolescent support and relatedness with classmates) and aggression toward peers while following adolescents over two courses of secondary school.

### 1.1. Social Relationships at School

In their increasing search for autonomy from their parents, adolescents’ school-based relationships, such as peer and teacher–student relationships, become highly influential not only for their academic adjustment but also for their psychosocial adjustment [1,9]. For example, students who are more liked by their peers or who are more popular seem to be happier [10], and students who report better relationships with teachers evidence higher self-esteem and prosocial behavior, and fewer depressive and problem behaviors [11,12]. It is important to note that many prior studies on social relationships at school have focused on social support perceived in the teacher–student relationship and on sociometric status among peers and, interestingly, research has found that both of these school-based relationships influence each other [13,14,15].

However, prior research on school-based relationships has paid limited attention to relatedness [16]. Relatedness, within the Self-Determination Theory (SDT) framework, is conceptualized as one of the three basic human needs (autonomy, competence, and relatedness) for well-being [17], and it is understood as a need to establish and maintain positive, meaningful, and enduring relationships. The construct of relatedness refers to the feeling that one is close and connected to significant others, and it is posited that positive adjustment will flourish in contexts where students feel that they care for and are cared for by key school figures such as teachers or peers [18]. Thus, an important task of educational research is to identify the determinants of student’s sense of relatedness in the classroom context—that is, to identify the contextual characteristics that support the fulfillment of this basic psychological need. This issue has not been completely addressed in the SDT literature [19,20].

In sum, previous research suggests a link between the quality of teacher–student and peer relationships, but the partial and cross-sectional nature of most past studies makes it difficult to attribute directionality to these relationships. Studies analyzing both school-based relationships simultaneously are scarce, even more so from an SDT perspective. From this theoretical framework, perceived teacher characteristics (such as perceived teacher support) could facilitate or block relatedness need satisfaction with classmates. The present study addresses this gap in the literature by examining the transactional relationships between teacher support and relatedness with classmates.

### 1.2. Social Relationships as Predictors of School Aggression

There is a general consensus in considering negative social relations at school with classmates and teachers as risk factors for the development of aggressive behaviors among adolescents [21,22]. For instance, it has been shown that low social preference (or peer rejection) can predict future aggression e.g., [15,23], and this, in turn, can be related to a status improvement [24]. Research has also revealed that low perceived support from teachers or perception of low teacher involvement is related to high aggression [12,25,26]. However, studies generally focus either on the influence of peers or of teachers, but studies involving the influence of both social agents on school aggression are scarce and show inconsistent results; see, for instance, [13,15]. In parallel, previous studies have highlighted that although relationships with peers and teachers are important on their own, a complete understanding of their contributions to students’ school adjustment requires the examination of their joint effects [2,27]. Furthermore, longitudinal studies are scarce e.g., [15], and have not considered the continuity and discontinuity of school-based relationships across successive academic courses, when the same group of classmates has a different teacher each year, which is typical in the Spanish secondary educational system.

Moreover, few studies have focused on the effects of relatedness in school aggression within an SDT perspective. Most of the studies applying the SDT perspective at school have found evidence for a positive effect of relatedness on academic engagement, performance, and well-being [2,16,28] but have not explored behavioral outcomes. In studies conducted with children and adolescents, findings indicate that unsatisfied psychological needs are linked to risk factors for adolescent aggression, such as lack of self-confidence [17] and negative emotions [29]; and that satisfied psychological needs are linked to prosocial behavior [30]. The few studies that have focused on peer aggression have informed that parental strategies that block adolescents’ autonomy positively predicted adolescents’ self-reported relational aggression [31,32]. However, none of these studies have focused on relatedness with classmates nor have they tested the SDT hypothesis of the mediational role of this variable between teacher support and school aggression.

### 1.3. Social Consequences of School Aggression

As seen, school aggression has relevant social origins but, simultaneously, empirical evidence also supports the social consequences of school aggression. Within a bio-ecological framework, it is relevant to focus on “children’s characteristics (such as aggression) which are features that invite or discourage reactions from the social environment that can disrupt or foster processes of psychological growth” [33] (p. 796). Aggressive behavior negatively affects the teaching and learning process, classroom climate, and teachers–students’ relationships [34]. In sociometric studies, one of the best predictors of being rejected by peers is a child’s aggressive behavior e.g., [35,36]. In adolescents, school aggression has been negatively related to a low perception of friendship in the classroom and positively to loneliness [8,32]. In addition, the presence of aggressive behaviors in the classroom is related to more negative teacher–student interactions [22] in terms of low closeness and perceived support, and high conflict [14,37,38].

Furthermore, findings indicate a transactional relationship between aggression and social consequences. For example, in sociometric longitudinal studies, children’s externalizing behavior consistently predicted low social preference the following year, and in turn, the experiences of low social preference added to the development of externalizing behavior [15,39]. The same has been found concerning the teacher–student relationship [14]. This supports the entanglement of peer social preference, teacher–child relationship, and externalizing behavior during school age, within a negative reinforcement cycle [40]. In effect, from a bio-ecological framework, researchers have reported that proximal social relationships (e.g., with peers and teachers) may not only influence children’s adjustment but may also predict each other throughout development e.g., [41]. In our longitudinal study, we contribute to the field by examining how school-based social relationships and school aggression relate to each other over time. More specifically, we seek to understand the role of aggression in shaping adolescent’s perceived teacher support and relatedness with classmates simultaneously.

### 1.4. Overt and Relational Aggression and Gender Differences

Traditionally, the scientific literature has extensively studied the problems arising from overt aggression (physical or verbal direct behaviors) at school [42]. However, more recent studies have been also interested in relational aggression and have shown that the more covert, subtle, and indirect forms of school aggression such as social exclusion, ostracism, and spreading false rumors may be as harmful to adolescent psychosocial adjustment as more overt and direct forms [43,44]. Two relevant issues can be added that justify the study of both types of aggression in adolescents. On the one hand, a differential perception has been observed in the two forms of aggression by adults, who tend to consider relational aggression as more acceptable than overt aggression, particularly during the stage of adolescence [45,46]. On the other hand, when comparing studies conducted with longitudinal data in Spanish schools (between 1999 and 2006), a decrease in behaviors involving overt aggression in adolescents, but not in behaviors of relational aggression was observed [47].

Moreover, the first studies that differentiated direct and indirect forms of school aggression were related to gender differences. These studies concluded that boys used direct aggression to a greater extent [42]. However, in the case of indirect aggression, more recent studies on gender differences have not been conclusive. For example, the review by Card and collaborators [45], a meta-analysis conducted with 148 studies, concludes that whereas there were significant gender differences in favor of boys in the use of direct forms of aggression, the differences in indirect aggression were minimal. These authors have argued that in adolescents, indirect aggressive strategies are yet fully developed for both genders.

The issue of possible gender differences in the causes and social consequences of the different forms of aggression has not received much attention. In sociometric studies, a relationship of both open and relational aggression and peer rejection in both genders has been observed as well as a positive relationship between relational aggression and peer acceptance in boys [48]. However, other authors have found two-way relationships between relational aggression and popularity in girls but not in boys, in whom popularity predicted greater relational aggression but not the contrary [49]. Concerning gender differences in social relationships with teachers and peers, it is important to note that girls generally perceive higher levels of emotional support from their teachers and school-based relatedness than boys [28,37,38,50]. Birch and Ladd [51] have argued that boys are more likely to disrupt class, and this in turn can to lead to more negative interactions with teachers. It is interesting to note that despite some gender differences in the mean-level of externalizing behavior, teacher–child relationships, and peer relationships, some studies have shown that there are no gender differences in the associations between these variables [14,15]. Therefore, bearing in mind previous research, it is interesting to analyze whether or not direct and indirect aggression are similar in their social origins and consequences for boys and girls.

### 1.5. The Present Study

Given the importance of school-based social relationships with adults and peers in adolescence and of aggression as a common problem in the schools of many countries, in this study, we extend earlier research by testing bidirectional associations between relationships with peers and teachers and school aggression in a longitudinal study across two courses of secondary school. We consider two forms of school aggression (overt and relational) as well as social support and relatedness as measures of school-based relationships with teachers and classmates, respectively. We were particularly interested in examining how both forms of aggression are differentially related to teacher support and relatedness over time. It is important to note that in Spain, with few exceptions, middle-school students (11 to 16 years) enter as a cohort in the first year and remain with the same classmates throughout secondary studies. In parallel, at the start of each school year, they are assigned a homeroom teacher who is in charge of taking care of students’ academic and personal-related affairs. To take into account the potential transactional links between both school-based relationships and aggressive behavior, a full cross-lagged longitudinal design was applied across two successive academic courses, which permits the examination of reciprocal effects between teacher–child and peer relationships while taking into account the previous levels of each variable and within-time associations between variables.

On the basis of the theoretical and empirical background, we can expect to find the following: (a) reciprocals effects between the two dimensions of school-based relationships (i.e., perceived teacher support and relatedness with classmates); however, consistent with the SDT framework [17], we expected stronger evidence for effects from teacher toward peers than vice versa; (b) a mediational effect of relatedness between perceived teacher support and aggression consistent with the SDT perspective [17]; (c) a mediational effect of relatedness between aggression and teacher support based on the bio-ecological framework [33,41]; and (d) stronger protective effects of teacher support for overt aggression than for relational aggression due to greater adult tolerance of this last form of aggression in adolescence [45,46].

Regarding gender differences, our hypotheses are less straightforward. Even though some previous studies found no gender differences in the pattern of associations between related variables [14,15], empirical evidence has shown consistent differences in the level of these variables for boys and girls (higher levels of perceived teacher support and relatedness, and lower level of overt aggression in girls). Hence, we reasoned that as causes, teacher support and relatedness with classmates could have stronger and protective effects against both forms of aggression in girls [37,50]. Concerning gender differences in the social consequences of aggression, some authors indicated worse consequences of relational aggression for girls [43], but others found social benefits for boys and girls [49]. Therefore, there is no consistent previous evidence for this last issue, and we only had a gender exploratory objective.

## 2. Materials and Methods

### 2.1. Participants

A total number of 826 students attending classes in 1st, 2nd, 3rd, and 4th grades of secondary education from five secondary schools participated in the study. All schools were located in urban areas in the northeast of Spain. The participants were evaluated in three waves: first, at the beginning of the academic year (T0), second, at the end of the same academic year (T1), and third, at the beginning of the next academic year (T2). For 172 students, data from questionnaires were missing two or more waves (hereafter, called dropout group), resulting in a three-wave longitudinal sample of 654 adolescents who participated in at least two waves. Dropout was due to finishing secondary education. The dropout group was eliminated from the study. The mean age of the adolescents in our study sample was 13.98 years (SD = 1.41) at T0, with ages ranging between 11 and 17; 22.6% of them were in first grade, 18.8 % were in second grade, 32.2% were in third grade, and 26.4% were in fourth grade, and 48% were boys. Background information was provided by 97% of the adolescents in our study sample. Of the families, 88.8% were intact, 9% were single-parent families, and 2.2% of the adolescents lived with other family members or non-family adults. Concerning education, 7.5% of the mothers and 14% of the fathers had completed higher education, 24.3 of the mothers and 24.6% of the fathers had completed high school, 26.2% of the mothers and 26.5% of the fathers had completed primary school, and 5% of the mothers and 4.4% of the fathers had no studies. The adolescents from the dropout group did not differ significantly from our study sample in age or gender at T0, and regarding study variables (teacher support, relatedness, overt aggression, and relational aggression) at any of the three times (detailed results of these analyses are available from the authors upon request).

### 2.2. Procedure

We contacted the principal of each school to explain the purpose of the research and to request permission to carry out the study. Then, we requested parents’ and guardians’ consent for the students to participate in this study. We explained the goals of the study to the students and informed them that participation was voluntary and anonymous. At least one qualified researcher (with a Ph.D.) was present during the administration of the instruments to provide students with the necessary support. We collected the same measures three times, separated by a 5-month interval. Measures were collected in all classrooms within 2 weeks at each time. The research was conducted in compliance with ethical values required for research on human beings, respecting the basic principles included in the Helsinki Declaration and the code of good practice in research of the host university. The research was also approved by the Vice-Chancellor of Scientific Policy of the University of Zaragoza (title of the study: Satisfaction of basic psychological needs, emotional regulation and aggression towards peers in adolescence, with protocol number JIUZ-2017-SOC-04).

### 2.3. Instruments

Data were collected to assess students’ perception of teacher support, relatedness with classmates, and aggression at school. Reliabilities of all measures were examined computing McDonald’s [52] omega based on factor loadings, after conducting a confirmatory factor analysis (CFA) using the weighted least square mean and variance adjusted (WLSMV) estimation method. Construct validity was tested using CFA.

#### 2.3.1. Teacher Support

We used the Interpersonal Relationships’ dimension of the Classroom Environment Scale (CES) [53], Spanish version of Fernández-Ballesteros and Sierra [54], which assesses the perception of the amount of help, trust, and friendship teachers offer to students in the classroom (e.g., “Teachers show particular interest in their students”). Response options ranged from 1 (strongly disagree) to 7 (strongly agree). Previous works e.g., [13,25] and the present study have shown evidence of reliability (omega values from 0.79 at T_1_ to 0.85 at T_2_).

#### 2.3.2. Relatedness

Students completed the five items of the Psychological Needs Scale [55], Spanish version by León et al. [56], regarding student’s relatedness. These items measured the degree of need satisfaction to maintain positive, meaningful, and enduring relationships with classmates (e.g., “I feel appreciated and valued by my classmates”) and were evaluated on a Likert scale ranging from 1 (strongly disagree) to 7 (strongly agree). Previous works [56] and the present study have shown evidence of reliability (omega values from 0.77 at T_0_ to 0.86 at T_2_).

#### 2.3.3. School Aggression

We used the Aggression Scale of Little, Henrich, Jones, and Hawley [57] adapted into Spanish by Estévez et al. [13]. Adolescents indicated the frequency with which they had engaged in aggressive behaviors toward peers at school over the last 12 months, on a seven-point scale from 1 (never) to 7 (many times). We used two scales to measure two dimensions of aggression: overt aggression (4 items, e.g., hitting others) and relational aggression (4 items, e.g., spreading rumors). Previous works [8,13,27] and the present study have shown evidence of reliability (omega values from 0.81 at T0 to 0.82 at T2 for overt aggression and from 0.71 at T0 to 0.84 at T2 for relational aggression).

### 2.4. Data Analyses

We began by computing mean, standard deviations, and correlations among major variables and testing mean differences across gender in the three waves of data. Then, to study the longitudinal reciprocal relations of perceived teacher support with relatedness and aggression (overt or relational), multivariate autoregressive cross-lagged modeling with three measurement waves was used [58]. With this kind of model, in comparison with a cross-sectional model, we can make causal claims because causes are assessed before mediators, and the mediators are, in turn, measured before outcomes [59]. Since we are interested in two forms of aggression (relational aggression and overt aggression) and in differences between girls and boys, we tested four models: the first two with the variables Teacher Support, Relatedness, and Overt Aggression in boys and girls, and the next two with Teacher Support, Relatedness, and Relational Aggression also in boys and girls.

We conducted analyses in Mplus 8 [60] using a Maximum Likelihood estimator. Maximum Likelihood requires data to be independent and normal. However, in this research, students were selected from several schools; thus, the observations might not be independent. To gather evidence of non-independence, we estimated the intraclass correlation (ICC); that is, the proportion of the total variance due to the group level versus the individual level. Values close to 1 indicate that the variable is completely group-dependent, whereas values close to 0 are an indicator that the variability is due to the subjects and not to the group. Since ICC values indicated that data were not independent (see Table 1), to avoid chi-square inflation and standard errors underestimation in the Structural Equation Model (SEM), we used the correction for non-independence (Type = complex in MPlus).

In addition to the non-independence of our data, the indicators in our model were items from Likert scales. These items had seven categories; thus, our data were ordered-categorical and non-normal. However, Rhemtulla et al. [61] showed that with six or seven categories, maximum likelihood with corrections for non-normality [62] produces acceptable results (Estimator = MLR in Mplus).

To diminish model complexity and allow comparison between waves, we constrained loadings of the same item on each major variable to the same value across the three waves. Finally, to analyze the mediational role of relatedness, we computed the indirect effect and its standard error using the delta method [63]. The Full Information Maximum Likelihood (FIML) was used to take account for missing data. To minimize possible biases, this method uses model covariates to predict missing data. FIML has been shown to be computationally efficient and superior to traditional strategies (i.e., listwise or pairwise) [64]. FIML produces less biased results than traditional strategies because it includes all available data in the estimation procedure [65].

## 3. Results

### 3.1. Descriptive Statistics and Mean Differences across Gender

Mean values and standard deviations are shown in Table 1. Means varied between 1.76 (overt aggression at T0) and 5.58 (relatedness at T0), and standard deviations varied between 0.85 (relational aggression at T0) and 1.23 (teacher support at T0). Correlations ranged from 0.083 (teacher support at T1 and relational aggression at T1) to 0.700 (relational aggression at T1 and overt aggression at T1).

Table 2 represents means and standard deviations of the study variables at the three measurement waves for girls (*N* = 342) and boys (*N* = 312). We found significant gender differences as follows: Girls showed, on average, a higher relatedness need satisfaction than boys at T1, *t*(653) = 2.390, *p* < 0.01, and at T2, *t*(653) = 2.597, *p* < 0.01, whereas boys perceived more teacher support at T1, *t*(653) = 1.645, *p* < 0.05, and at T2, *t*(653) = 2.045, *p* < 0.05. In addition, compared to girls, boys had significantly higher mean levels of both types of aggression, overt and relational, at each measurement wave: *t*(653) = 6.053, *p* < 0.001 for overt aggression and *t*(653) = 4.692, *p* < 0.001 for relational aggression at T0; *t*(653) = 5.600, *p* < 0.001 for overt aggression and *t*(653) = 4.485, *p* < 0.001 for relational aggression at T1; *t*(653) = 4.777, *p* < 0.001 for overt aggression and *t*(653) = 4.670, *p* < 0.001 for relational aggression at T2.

### 3.2. Cross-Lagged Models

#### 3.2.1. Overt Aggression

**Figure 1:**
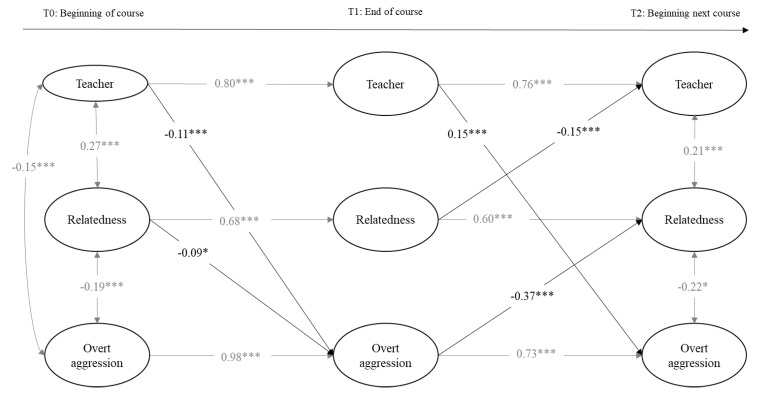
Cross-lagged model predicting longitudinal reciprocal relations between teacher support, relatedness, and overt aggression in girls. Only estimates significant at *p* < 0.05 or less are provided. Cross-lagged paths are depicted in bold. * *p* < 0.05; *** *p* < 0.001.

**Figure 2:**
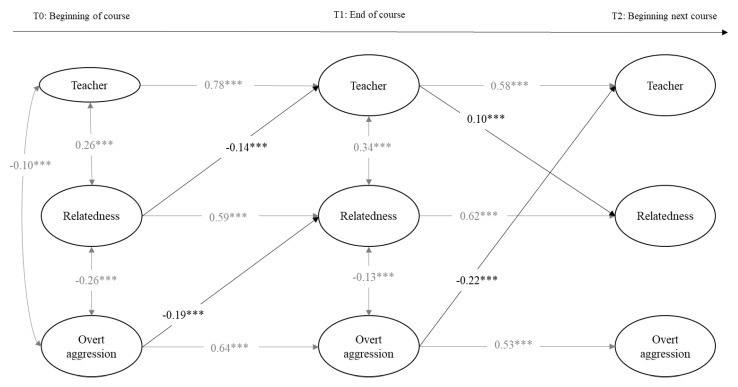
Cross-lagged model predicting longitudinal reciprocal relations between teacher support, relatedness, and overt aggression in boys. Only estimates significant at *p* < 0.05 or less are provided. Cross-lagged paths are depicted in bold. *** *p* < 0.001.

The two first calculated models included the variables of perceived teacher support, relatedness, and overt aggression toward peers in girls and boys separately. Model fits were adequate: for girls, the chi-square values and the fit indexes were χ^2^(342, 814) = 978.35, *p* = 0.00, RMSEA (Root Mean Square Error of Approximation) = 0.02 [0.02, 0.03], CFI (Comparative Fit Index) = 0.95, and TLI (Tucker Lewis Index) = 0.95; and for boys, they were χ^2^(312, 814) = 956.5, *p* = 0.00, RMSEA = 0.02 [0.02, 0.03], CFI = 0.97, and TLI = 0.97. As can be seen in Figure 1 and Figure 2, teacher support, relatedness, and overt aggression were stable over time within the same academic course (T0 and T1) and across the beginning of the next academic course (T2). Within T0, in boys and girls, teacher support and relatedness were positively associated, and overt aggression was negatively related to teacher support and relatedness. In addition, relatedness was positively related to teacher support and negatively to overt aggression at T1 only in boys and at T2 only in girls. Above and beyond the stability paths and within the time associations, cross-time associations were found. Regarding associations between relatedness and teacher support, relatedness negatively predicted teacher support from T1 to T2 in girls. In boys, relatedness negatively predicted teacher support from T0 to T1 in boys, which, in turn, positively predicted relatedness at T2. With regard to the associations with overt aggression in girls, teacher support negatively predicted overt aggression from T0 to T1, but this association was positive from T1 to T2. Moreover, from T0 to T1, relatedness negatively predicted overt aggression, which, in turn, negatively predicted relatedness in T2. In boys, overt aggression negatively predicted relatedness from T0 to T1 and teacher support from T1 to T2.

Concerning the indirect effects in the overt aggression models, we calculated all of them derived from previous significant cross-time associations. As presented in Table 3, we found evidence for multiple significant indirect effects over time involving two of the three study variables, but we only gathered evidence of a significant indirect effect involving the three study variables over time in girls: teacher support at T0 was positively related to relatedness at T2 through a decrease in overt aggression at T1. Indirect effect values varied between −0.01 (relatedness at T0 was negatively related to relatedness at T2 through a decrease in teacher support at T1 in boys) and −0.36 (overt aggression at T0 was negatively related to relatedness at T2 through continuity of overt aggression at T1 in girls).

#### 3.2.2. Relational Aggression

**Figure 3:**
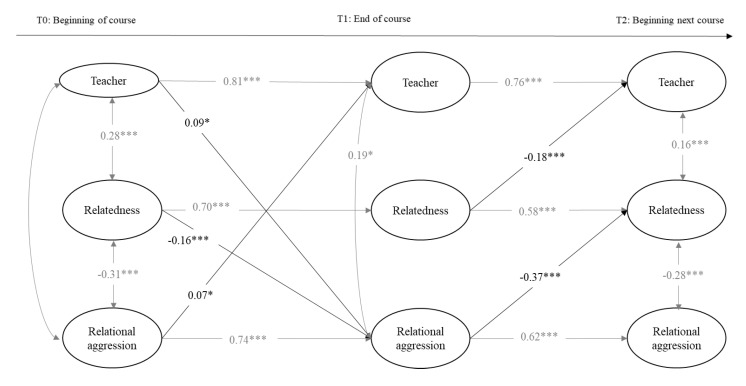
Cross-lagged model predicting longitudinal reciprocal relations between teacher support, relatedness, and relational aggression in girls. Only estimates significant at *p* < 0.05 or less are provided. Cross-lagged paths are depicted in bold. * *p* < 0.05; *** *p* < 0.001.

**Figure 4:**
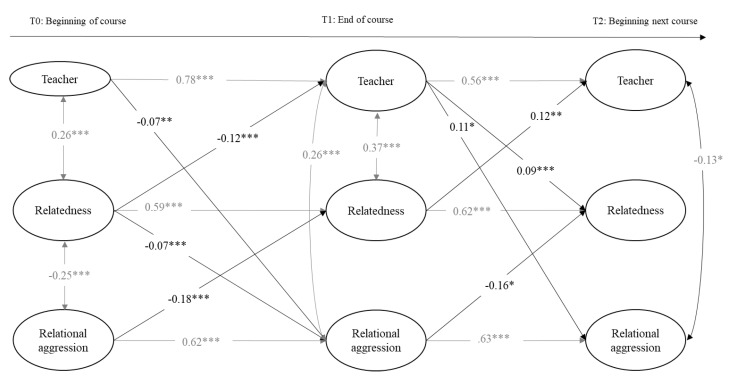
Cross-lagged model predicting longitudinal reciprocal relations between teacher support, relatedness, and relational aggression in boys. Only estimates significant at *p* < 0.05 or less are provided. Cross-lagged paths are depicted in bold. * *p* < 0.05. ** *p* < 0.01. *** *p* < 0.001.

The next two calculated models included the variables of perceived teacher support, relatedness, and relational aggression toward peers in girls and boys separately. Model fits were adequate: for girls, the chi-square values and the fit indexes were *χ*^2^(342, 814) = 977.17, *p* = 0.00, RMSEA = 0.02 [0.02, 0.03], CFI = 0.95, and TLI = 0.95; and for boys, they were *χ*^2^(312, 814) = 933.96, *p* = 0.002, RMSEA = 0.02 [0.01, 0.03], CFI = 0.97, and TLI = 0.97. As can be seen in Figure 3 and Figure 4, teacher support, relatedness, and relational aggression were stable over time within the same academic course (T0 and T1) and at the beginning of the next academic course (T2). Within T0 and T1, in boys and girls, teacher support and relatedness were positively associated, and relatedness and relational aggression were negatively associated. In addition, teacher support and relational aggression were negatively associated at T0 in girls and at T2 in boys. This association was positive at T1 in girls and boys. In addition, teacher support and relatedness were positively associated in boys. Above and beyond the stability paths and within the time associations, cross-time associations were found: regarding associations between relatedness and teacher support, relatedness negatively predicted teacher support from T0 to T1 in boys and from T1 to T2 in girls, but this association was positive from T1 to T2 in boys. In addition, teacher support positively predicted relatedness from T1 to T2 in boys. Concerning the associations with relational aggression in girls, teacher support positively predicted relational aggression from T0 to T1 and, inversely, relational aggression positively predicted teacher support from T0 to T1. From T0 to T1, in both genders, relatedness negatively predicted relational aggression, which in turn negatively predicted relatedness at T2. In boys, teacher support and relatedness negatively predicted relational aggression from T0 to T1, but the association between teacher support and relational aggression from T1 to T2 was positive.

Concerning the indirect effects in the relational aggression models, we calculated all of them derived from previous significant cross-time associations. As presented in Table 4, we found evidence for multiple significant indirect effects over time involving two of the three study variables, but we only gathered evidence of a significant indirect effect involving the three study variables over time in boys: relational aggression at T0 was negatively related to teacher support at T2 through a decrease in relatedness at T1. Indirect effect values varied between −0.01 (relatedness at T0 was negatively related to relatedness at T2 through a decrease in teacher support at T1 in boys) and −0.27 (relational aggression at T0 was negatively related to relatedness at T2 through continuity of relational aggression at T1 in girls).

## 4. Discussion

The main objective of this study was to analyze the transactional links between social school-based relationships (teacher–adolescent support and relatedness with classmates) and aggression toward peers when following adolescents over two courses of secondary school. We tested the pattern of associations between the study variables in two forms of aggression (relational aggression and overt aggression) and in boys and girls separately. Findings contribute to the field by showing how school-based social relationships and these two school forms of aggression relate to each other over time. In this section, we discuss cross-time associations between variables considering gender differences transversely.

First of all, we tested if the level of the study variables was different in boys and girls. In accordance with previous studies, in our results, girls showed a higher relatedness need satisfaction compared to boys [28,37]; but, contrary to previous literature [36,48], in our results, boys perceived more support from their teachers than girls. Another gender difference obtained points to a consistently higher amount of aggression, both overt and relational, exhibited by boys compared to girls at each measurement wave. These results add evidence in favor of the higher prevalence of overt aggression in boys [45] and expand the previous evidence in relational aggression also in favor of boys [57].

### 4.1. Social Relationships at School

In our first specific objective, we intended to analyze the directionality of the relationships between the two dimensions of school-based relationships (perceived teacher support and relatedness with classmates), expecting, from an SDT perspective, stronger evidence for a positive effect of perceived teacher’s characteristics (perceived teacher support) on students’ relatedness need satisfaction than vice versa. Our results suggest evidence for both directions in boys but not in girls. In boys, it seems that perceived teacher support over the academic course promotes relatedness with classmates at the beginning of the next course and vice versa; a good relatedness with classmates during the course is related to higher perceived teacher support at the beginning of the next course. Thus, in boys, the two school-based relationships positively influence each other. This result is consistent with previous literature measuring sociometric status and perceived teacher support [13,14,38]. However, in girls, a good relatedness with classmates during the course is related to lower perceived teacher support at the beginning of the next course. It is possible that girls are more oriented toward the development of meaningful relationships with their peers [66], and when their relatedness needs with classmates are satisfied, they may not need and therefore may not perceive the support of their teachers. It could also be possible that teachers may offer less support to girls who have good relatedness with classmates. Concerning this result, it is interesting to note that in their recent study, León and Liew [2] have shown that low responsiveness from the teacher does not necessarily lead to poor school outcomes (low grades or well-being) if students have at least moderate feelings of relatedness with classmates. It seems that when comparing support from different school sources, peer support is more relevant that teacher support in predicting some positive school outcomes [9]. More research is needed to explain these results and gender differences.

### 4.2. Social Relationships as Predictors of School Aggression

Secondly, we analyzed the influence of relationships with both social agents (peers and teachers) simultaneously in school aggression. Specifically, we expected a mediational effect of relatedness with classmates between perceived teacher support and aggression, which is consistent with an SDT perspective. Our results partially support this hypothesis. On the one hand, we found a protective effect of relatedness against relational aggression in both genders: relatedness at the beginning of the course predicted lower relational aggression at the end of the course and the beginning of the next course. Moreover, relatedness at the beginning of the course predicted relatedness at the beginning of the next course through a decrease of relational aggression at the end of the course. These results are coherent with previous sociometric literature [15,23] and the SDT perspective [17], and they expand previous evidence about benefits of the satisfaction of the relatedness need, as a basic psychological need, from school outcomes such as academic achievement, engagement, and well-being [2,16,28] to behavioral outcomes. Boys and girls who start the course with high satisfaction of their need for relatedness with their classmates do not engage in relational aggression behaviors throughout the course or at the beginning of the next course, which also benefits their later level of relatedness. Moreover, these results were stronger for girls than for boys (the beta coefficient of indirect effects was more than twice as high in girls as in boys), adding partial evidence (only for relational aggression) to our gender hypothesis that relatedness could have stronger and protective effects against aggression in girls than in boys [37,50].

On the other hand, we found a protective effect as well as a risk effect of perceived teacher support for school aggression. Interestingly, we observed a protective effect against aggression in the medium (end of the course) and long term (beginning of the next course) when support was only provided initially (at the beginning of the course). This result is coherent with previous evidence supporting the protective effect of positive relationships with the teacher against school aggression and problem behavior in general terms [12,27]. Moreover, we also found that initial perceived teacher support was beneficial for relatedness with classmates at the beginning of the next course through a decrease in overt aggression in girls. However, teacher support was a risk for long-term aggression (at the beginning of the next course) when the teacher’s perceived support was maintained throughout the course. It seems that in this last case, the continuity of teacher support can act as a reinforcement for aggressive students [67]. To prove this hypothesis, it would be necessary to complement studies with specific naturalist observations to analyze in detail aggressive student–teacher interactions throughout a course.

These two teacher support effects were observed in boys and girls, but gender differences were found in the form of aggression analyzed: teacher support, as an initial protective factor and a risk factor if maintained, was related to overt aggression only in girls and to relational aggression only in boys. According to “gender normative forms of aggression” [48], perceived teacher support might act as a protective or a risk factor only for adolescents displaying a gender-atypical form of aggression. Adolescents who behave in an unexpected gender way for adults’ stereotypic expectations may attract teachers’ attention and, as a consequence, perceive more support from them, and this increment in perceived teacher support could act as an initial protection against aggression or as a positive reinforcer of the “unexpected behavior” if maintained throughout the course. These results partially support our fourth hypothesis, as we expected stronger protective effects of teacher support for overt aggression than for relational aggression due to greater adult tolerance of this latter form of aggression in adolescence [45,46]. However, we clarify that this only occurs when support is perceived at the beginning of the course (and not maintained throughout the course) and concerning gender-atypical forms of aggression that could be more “visible” to adults.

### 4.3. Social Consequences of School Aggression

Third, we also analyzed the social consequences of school aggression in terms of costs for the relationships with peers and teachers. We found that relational aggression in boys at the beginning of the course was related to a decrease in perceived teacher support at the beginning of the next course through a decrease in relatedness at the end of the course. That is, relational aggressive behaviors at the beginning of a course have medium-term interpersonal costs with peers (less relatedness) and long-term interpersonal costs with the teacher (less perceived support). This mediational effect supports the entanglement of school aggression, relatedness with classmates, and the teacher–student relationship within a negative reinforcement cycle [40]. As explained previously, in Spain, students remain with the same classmates throughout secondary education, but they are assigned to a new homeroom teacher each school year. Hence, our results point to a kind of expectation transmission [68] about problematic students among the teaching staff that could be affecting the new homeroom teacher–student supportive relationships at the beginning of the new course.

Finally, our results showed relevant negative consequences of aggression on school relationships at the peer level: girls who engaged in overt and relational aggression throughout a course showed less relatedness with classmates at the beginning of the next course, and boys who were overtly aggressive at the beginning of the course showed less relatedness with classmates both at the end of the course and the beginning of the next. These results are in line with previous sociometric literature pointing to the social costs of aggression in terms of low social preference or peer rejection [36]. Moreover, considering some studies that have reported social benefits (popularity) of physical aggression [14] and relational aggression [49], our results go a step further by suggesting that these alleged benefits could be more apparent than real because, subjectively, aggressive adolescents show a low level of relatedness with classmates. Overall, our results show worse social consequences of overt and relational aggression for girls than for boys (all observed indirect effects were higher in girls than in boys). In sum, these results provide a deeper analysis of the social consequences of school aggression from the adolescent’s point of view.

### 4.4. Limitations and Future Directions

Some limitations of the study should be mentioned. Firstly, we only gathered information via anonymous questionnaires; thus, complementing the study with other informants (teacher and peer reports) and other instruments (naturalist observation) could lead to a broader and more comprehensive approach to the topic, especially for the analysis of the potential reinforcement of aggressive behavior through maintained teacher support. Secondly, as explained above, in Spanish secondary schools, passing from one course to the next involves changing teachers, whereas classroom composition mostly remains intact. We believe that this fact could inflate the influence of relatedness with classmates, as compared to teacher support, and limit the generability of the results to schools with less changing of homeroom teachers or less stable classroom composition from course to course. Third, previous studies have found a decrease in aggressive behavior toward peers as adolescence progresses, especially in overt aggression [69,70]. Future research should contrast these transactional links taking into account age groups and including late adolescence (17–20 years old).

## 5. Conclusions 

Despite these and other possible limitations, we highlight the contribution of these results to the field of school aggression. Our results provide a better understanding of profiles of students who may be at risk for relational and overt aggression at school (those adolescents who start the course with low levels of relatedness and perceived teacher support) but also a better understanding of the consequences of school aggression at a social level (poor relationships with classmates and teachers), with specific differential considerations for girls and boys. 

## 6. Implications for Practice

We observe that in the absence of an intervention, aggression does not decrease spontaneously. Indeed, in our results, boys’ and girls’ means in overt and relational aggression tend to increase over the time. Therefore, our results highlight the relevance of early interventions (at the beginning of a course) in classrooms with the presence of aggressive behaviors toward peers at a minimum of two levels.

First, by increasing teacher support perceived by students at the beginning of the course, we could decrease overt (especially in girls) and relational aggression (especially in boys) in the medium and long term. It would be necessary to follow up adolescents who do not reduce their aggressive behavior despite this early intervention. In those cases, it would be necessary to change intervention objectives because, in line with our results, it could be possible that the maintenance of high levels of teacher attention and support over the course could be reinforcing aggression. Interventions focused on teachers would have to include a gender perspective because we observed that protective and risk effects of teacher support occur only in gender-atypical forms of aggression that may be more unexpected for adults. However, both forms of aggression are exhibited both by boys and girls (with higher levels in boys) and need to be detected by teachers for efficient behavioral management. 

Second, at a classroom group level, interventions increasing the satisfaction of the relatedness with classmates at the beginning of the course could decrease relational aggression over the medium and long term with benefits for future relatedness, especially in girls. It is important to note that at this point, parallel interventions focused on teachers will be necessary to prevent the possible lack of involvement of teachers with girls who exhibit a good relatedness during a course or with boys who show relational aggression and poor relatedness during the previous course. In short, this study provides evidence arguing that an early intervention focused on developing harmony and good relationships between students and teachers at the beginning of the course will be a benefit in the face of the development of aggression toward peers and, therefore, for the psychological well-being and academic adjustment of adolescents.

## Figures and Tables

**Table 1:** Descriptive statistics, ICC values, and Pearson’s correlations.

Variables	M	SD	ICC	% Non Missing	1	2	3	4	5	6	7	8	9	10	11
1. Relatedness T0	5.58	0.87	0.00	0.98											
2. Relatedness T1	5.42	0.94	0.01	0.94	0.503 **										
3. Relatedness T2	5.39	1.02	0.00	0.63	0.469 **	0.508 **									
4. Teacher support T0	3.55	1.23	0.04	0.96	0.201 **	0.134 **	0.188 **								
5. Teacher support T1	3.39	1.15	0.06	0.94	0.094 *	0.150 **	0.103 *	0.583 **							
6. Teacher support T2	3.42	1.17	0.07	0.63	0.043	0.071	0.123 *	0.428 **	0.566 **						
7. Overt aggression T0	1.76	0.86	0.03	0.91	−0.120 **	−0.170 **	−0.314 **	−0.048	−0.009	−0.084					
8. Overt aggression T1	1.93	0.99	0.01	0.92	−0.109 **	−0.212 **	−0.208 **	−0.036	−0.043	−0.021	0.470 **				
9. Overt aggression T2	2.03	1.06	0.01	0.63	−0.094	−0.074	−0.230 **	−0.033	0.067	0.034	0.426 **	0.434 **			
10. Relational aggression T0	1.77	0.85	0.02	0.91	−0.120 **	−0.138 **	−0.280 **	−0.032	0.036	0.011	0.696 **	0.373 **	0.333 **		
11. Relational aggression T1	1.90	0.94	0.00	0.92	−0.143 **	−0.207 **	−0.182 **	−0.028	0.083 *	0.076	0.346 **	0.700 **	0.362 **	0.396 **	
12. Relational aggression T2	2.01	1.07	0.02	0.63	−0.084	−0.058	−0.242 **	0.011	0.134 **	0.087	0.339 **	0.371 **	0.836 **	0.387 **	0.413 **

Note: M = Mean; SD = Standard deviation; ICC = Intraclass correlation; % not missing = percentage of non-missing data; * *p* < 0.05. ** *p* < 0.01.

**Table 2:** Means and standard deviations at the three measurement waves for girls (*N* = 342) and boys (*N* = 312).

	Wave 1				Wave 2				Wave 3			
	Girls		Boys		Girls		Boys		Girls		Boys	
	*M*	*SD*	*M*	*SD*	*M*	*SD*	*M*	*SD*	*M*	*SD*	*M*	*SD*
Teacher support	3.53	1.38	3.58	1.67	3.31	1.24	3.46	1.4	3.32	1.16	3.51	1.6
Relatedness	5.59	0.77	5.58	0.73	5.50	0.90	5.33	0.87	5.49	0.85	5.28	1.24
Overt aggression	1.57	0.47	1.97	0.95	1.72	0.74	2.14	1.12	1.84	0.99	2.22	1.15
Relational aggression	1.63	0.54	1.94	0.88	1.74	0.66	2.07	1.08	1.82	0.96	2.20	1.23

**Table 3:** Indirect effects in the overt aggression model.

				95% CI	
Indirect Paths in Girls’ Model	*β*	SE	*p*	LL	UL
Teacher Support T0—Teacher Support T1—Overt Aggression T2	0.12	0.02	<0.001	0.08	0.16
Teacher Support T0—Overt Aggression T1—Relatedness T2	0.04	0.01	<0.001	0.03	0.05
Teacher Support T0—Overt Aggression T1—Overt Aggression T2	−0.08	0.01	<0.001	−0.09	−0.07
Relatedness T0—Relatedness T1—Teacher Support T2	−0.10	0.03	<0.001	−0.16	−0.05
Overt Aggression T0—Overt Aggression T1—Relatedness T2	−0.36	0.05	<0.001	−0.45	−0.27
**Indirect paths in boys’ model**					
Teacher Support T0—Teacher Support T1—Relatedness T2	0.08	0.01	<0.001	0.07	0.10
Relatedness T0—Teacher Support T1—Teacher Support T2	−0.08	0.02	<0.001	−0.14	−0.05
Relatedness T0—Teacher Support T1—Relatedness T2	−0.01	0.00	<0.001	−0.02	−0.01
Overt Aggression T0—Relatedness T1—Relatedness T2	−0.11	0.02	<0.001	−0.18	−0.10
Overt Aggression T0—Overt Aggression T1—Teacher Support T2	−0.14	0.04	<0.001	−0.26	−0.09

Note: β = standardized beta weight; SE = standard error; CI = confidence interval; LL = lower limit; UL = upper limit.

**Table 4:** Indirect effects in the relational aggression model.

				95% CI	
Indirect Paths in Girl’s Model	*β*	SE	*p*	LL	UL
Relatedness T0—Relatedness T1—Teacher Support T2	−0.12	0.03	<0.001	−0.17	−0.07
Relatedness T0—Relational Aggression T1—Relatedness T2	0.06	0.01	<0.001	0.03	0.09
Relatedness T0—Relational Aggression T1—Relational Aggression T2	−0.10	0.02	<0.001	−0.14	−0.06
Relational Aggression T0—Relational Aggression T1—Relatedness T2	−0.27	0.05	<0.001	−0.37	−0.17
**Indirect paths in boy’s model**					
Teacher Support T0—Teacher Support T1—Relatedness T2	0.08	0.01	<0.001	0.07	0.10
Teacher Support T0—Teacher Support T1—Relational Aggression T2	0.08	0.03	<0.05	0.02	0.15
Teacher Support T0—Relational Aggression T1—Relational Aggression T2	−0.04	0.02	<0.05	−0.08	−0.01
Relatedness T0—Teacher Support T1—Teacher Support T2	−0.07	0.02	<0.001	−0.10	−0.03
Relatedness T0—Teacher Support T1—Relatedness T2	−0.01	0.00	<0.001	−0.02	−0.01
Relatedness T0—Relatedness T1—Teacher Support T2	0.07	0.02	<0.01	0.03	0.12
Relatedness T0—Relational Aggression T1—Relatedness T2	0.01	0.00	<0.05	0.00	0.02
Relatedness T0—Relational Aggression T1—Relational Aggression T2	−0.04	0.01	<0.001	−0.06	−0.02
Relational Aggression T0—Relatedness T1—Teacher Support T2	−0.02	0.01	<0.01	−0.18	−0.10

Note: β = standardized beta weight; SE = standard error; CI = confidence interval; LL = lower limit; UL = upper limit.
